# Mesenchymal Stem Cells Combined with Hepatocyte Growth Factor Therapy for Attenuating Ischaemic Myocardial Fibrosis: Assessment using Multimodal Molecular Imaging

**DOI:** 10.1038/srep33700

**Published:** 2016-11-02

**Authors:** Huizhu Chen, Rui Xia, Zhenlin Li, Lizhi Zhang, Chunchao Xia, Hua Ai, Zhigang Yang, Yingkun Guo

**Affiliations:** 1 Department of Radiology, Key Laboratory of Obstetric & Gynecologic and Pediatric Diseases and Birth Defects of Ministry of Education, West China Second University Hospital, Sichuan University, China; 2 Department of Radiology, State Key Laboratory of Biotherapy, West China Hospital, Sichuan University, China; 3 National Engineering Research Center for Biomaterials, Sichuan University, China

## Abstract

Clinically, myocardial fibrosis is increasingly being recognized as a new therapeutic target for ischaemic heart diseases. The aim of this study was to investigate whether noninvasive multimodal molecular imaging could be used to dynamically assess whether the combination of bone marrow mesenchymal stem cells (BMSCs) and hepatocyte growth factor (HGF) therapy can synergistically attenuate myocardial fibrosis after myocardial infarction (MI). MI was induced in 28 rats by coronary ligation with subsequent injection of BMSCs/HGF, BMSCs, HGF, or saline into the border zone under echocardiography guidance. The therapeutic procedure and treatment effects were tracked and assessed using bioluminescence imaging (BLI) and cardiac magnetic resonance (MR) imaging. Four weeks after transplantation therapy, cardiac MR imaging demonstrated that BMSC/HGF-treated animals showed better ejection fractions (*p* < 0.001) and smaller scar sizes (*p* < 0.001) than those treated with BMSCs or HGF alone. Histopathological and immunohistochemical results showed less collagen deposition, increased microvessel densities and more regenerative cardiomyocytes in the BMSC/HGF-treated animals than in those receiving HGF or BMSCs alone (all *p* < 0.05). Multimodal molecular imaging allows a specific and timely strategy to be established for dynamically tracking treatment and noninvasively assessing the therapeutic effects. Under echocardiography guidance, intramyocardial injection of transfected HGF with BMSCs can enhance cell survival, improve cardiac function, stimulate angiogenesis, and reduce myocardial fibrosis in a post-MI rat model.

Myocardial fibrosis is a common pathological condition of the extracellular matrix, mainly affecting type I and III collagen remodelling in the myocardium. This condition often leads to myocardial stiffness, cardiac dysfunction, arrhythmia, and increased mortality^1^,^2^,^3^,^4^,^5^. Fibrosis is a hallmark of end-stage cardiac injury and the most common outcome in cases of ischaemic cardiomyopathy^2^. Currently, myocardial fibrosis is increasingly being recognized as a new therapeutic target for heart diseases^1^. After infarction, a feasible therapeutic strategy to attenuate left ventricular (LV) remodelling is to increase perfusion and function. Notably, early intervention can prevent disease progression and long-term fibrosis accumulation^3^. So far, the traditional drug treatments for myocardial fibrosis are ineffective, and cardiac transplantation is associated with many clinical risks. In view of this dilemma, regenerative medicine is a new and promising therapeutic choice for myocardial fibrosis^4^,^5^.

Previous studies^5^,^6^ have demonstrated that bone marrow mesenchymal stem cells (BMSCs) facilitate both cardiac function improvement and scar size reduction with high levels of safety and efficacy. The underlying therapeutic mechanisms of BMSCs primarily include differentiation into functional cardiomyocytes, paracrine effects, and stimulation of endogenous neovascularization^6^,^7^. Moreover, gene therapy is emerging as a potentially new treatment for ischaemic cardiomyopathy^8^. Data from previous studies^9^,^10^ have demonstrated that cardiac hepatocyte growth factor (HGF) is a potent angiogenic, anti-apoptotic, and anti-fibrotic agent for treating myocardial infarction (MI). Recently, the positive role of HGF in facilitating the differentiation of stem cells into cardiomyocytes has been increasingly recognized as a rational approach to improve the efficacy of MSC transplantation for MI^8^,^9^,^10^. Herein, we hypothesized that combined treatment with HGF and BMSCs can stimulate cardiac repair and attenuate myocardial fibrosis through synergistic effects. To test this hypothesis, we injected superparamagnetic iron oxide (SPIO) and luciferase double-labelled BMSCs transfected with lentivector-mediated human HGF into the border of the infarct zone 7 days after myocardial infarction under echocardiography guidance. The survival and differentiation of the injected BMSCs and their effects on cardiac restoration and myocardial fibrosis were monitored and assessed dynamically *in vivo* using bioluminescence imaging (BLI) and cardiac magnetic resonance (MR) imaging, as described in previous reports^11^,^12^,^13^. Moreover, HGF gene effects on infarcted myocardium have been demonstrated using Cine, perfusion and viability MR imaging^14^. Thus, the specific aim of our study was to investigate whether the combination of BMSCs and HGF therapy could synergistically improve cardiac function, stimulate endogenous neovascularization and attenuate myocardial fibrosis after MI. Furthermore, we confirmed a new strategy for noninvasive and dynamic monitoring of the therapy processes *in vivo* and for quantitatively assessing the treatment effects using multimodal molecular imaging tracer techniques.

## Results

### Molecular Imaging Tracking

**Figure 1:**
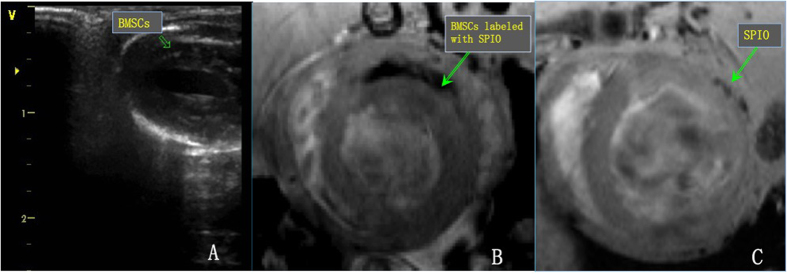
Bone mesenchymal stem cell (BMSC) tracking *in vivo*. (**A**) On transthoracic echocardiography, BMSCs at the border of the infarcted myocardium appeared as hyper-echoic lesions. On the cardiac MR imaging, SPIO-labelled BMSCs were visible as a hypo-intense area with sharp borders at the injection site after transplantation (**B**), but the SPIO signals became nearly undetectable after 14 days (**C**).

**Figure 2:**
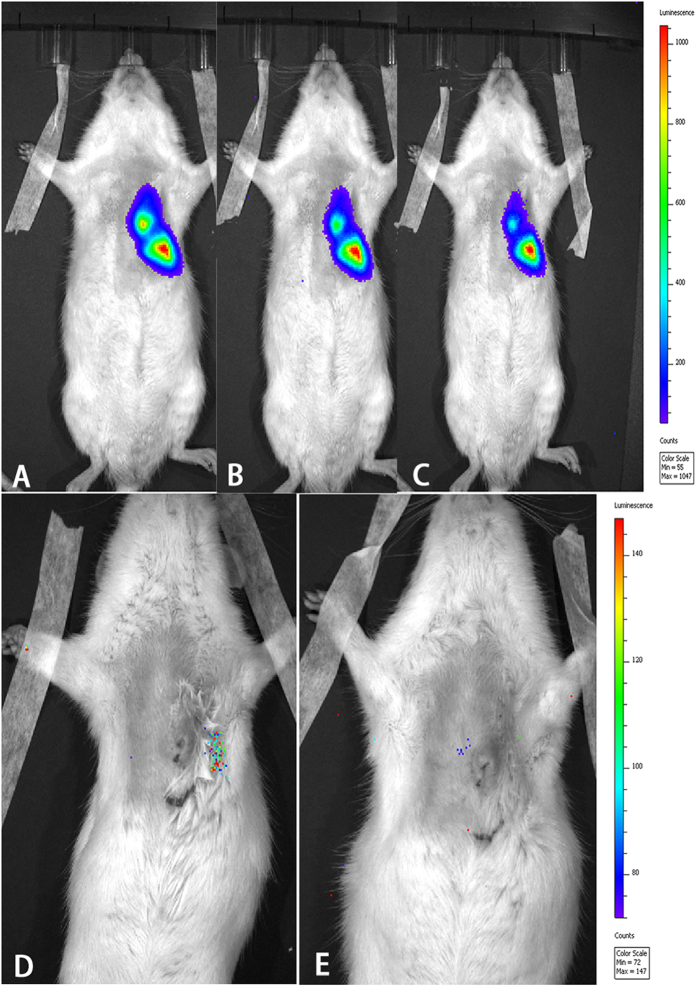
Bioluminescence imaging (BLI) dynamically monitored the survival status of the BMSCs. After intramyocardial injection, BLI was used to monitor the survival of transfected BMSCs *in vivo*. We found that transfected BMSC signals can be detected from 40 min (**A**) to 3 days (**B**), 5 day (**C**), and 7 days (**D**). However, the signals became undetectable after 9 days (**E**).

After successful double-labelling using luciferase and PEI2k-SPIO, the BMSCs and BMSCs/HGF were injected into the border of the infarct area under echocardiography guidance. Cells transplanted into the myocardium appeared as hyperechoic lesions on transthoracic echocardiography. On the cardiac MR imaging, all SPIO-labelled BMSCs were visible as a hypointense area with sharp borders at the injection site within a week after transplantation, but the SPIO signals became nearly undetectable after 14 days (Fig. 1). Bioluminescence imaging (BLI) was used to dynamically monitor the survival rate of BMSCs in the myocardium after intramyocardial injection. BLI/D-Luc signals were detectable 40 min after the D-Luciferin injection. Furthermore, BLI/D-Luc signals of the BMSC group declined gradually 3 days after injection and disappeared completely by day 7. However, we found that signals from the BMSCs/HGF group steadily appeared after 5 days and became undetectable after 9 days (Fig. 2).

### Cardiac Function

**Figure 3:**
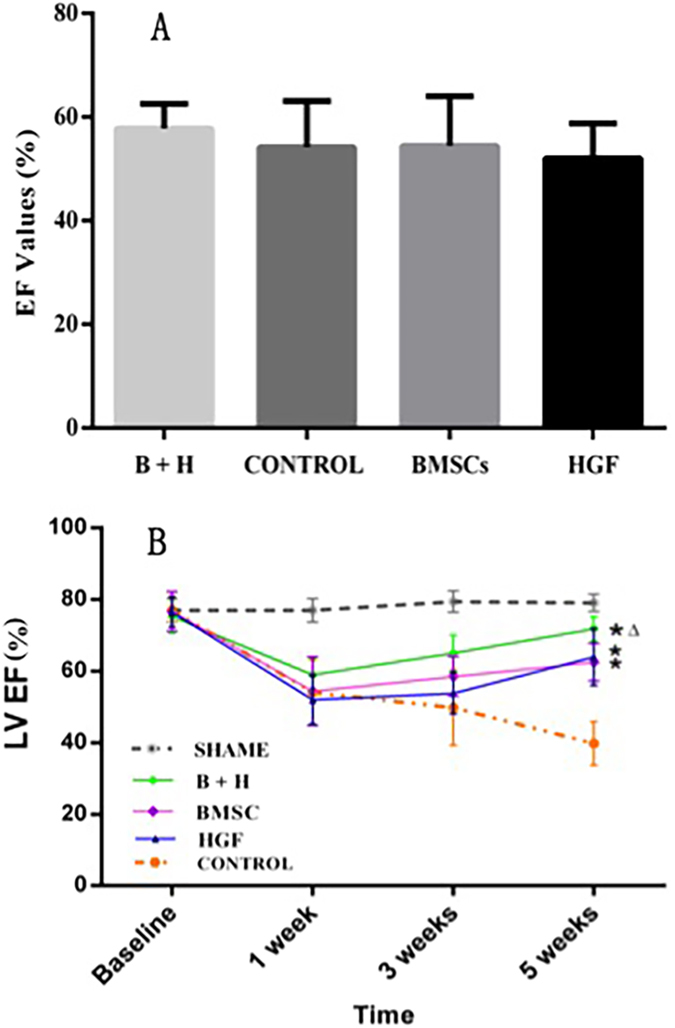
LV function after ligation was followed using cardiac MR imaging. (**A**) Baseline data showed no significant difference between pre-operation LVEF values among all the groups. (**B**) Before injection, no statistically significant differences in LVEF reductions were found among all treated groups and the control group (all *p* > 0.05) one week after MI. After injection, the LVEF of the control group continuously declined after ligation. LVEF was significantly increased in all treated groups (*p < 0.05 versus the control group), especially in the BMSCs/HGF treatment group (^△^*p* < 0.001).

Before surgery, the baseline data showed that the LVEF was not significantly different between the treated and sham groups (all *p* > 0.05). One week after MI, before injection, LVEF decreased markedly in all the groups. The absence of a statistically significant difference in LVEF reduction among these groups indicated that the infarction injuries were similar among the groups (all *p* > 0.05) (Fig. 3A). After infarction, LVEF continuously deteriorated during the 5-week follow-up period in control animals. By contrast, all the 3 treated groups showed significant improvement in LV function 5 weeks after MI. Although groups treated with HGF alone and BMSC alone had comparable LVEF improvements of 16.7 ± 5.4% (*p* < 0.001) and 12.9 ± 4.7% (*p* = 0.01) at 5 weeks after infarction, the combination of BMSCs and HGF produced a marked improvement in the ejection fraction of 24.5 ± 4.2% (*p* < 0.001) (Fig. 3B).

### Myocardial Fibrosis

**Figure 4:**
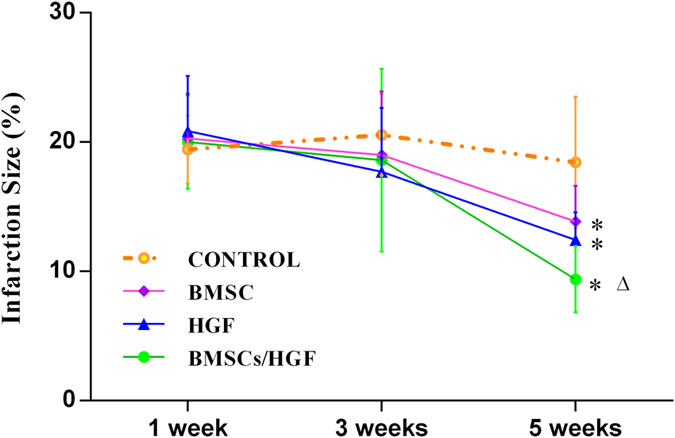
Infarct size after injection measured using MR imaging. The infarc sizes in all treated groups with chronic infarction after 5 weeks of MI were significantly smaller than acute MI except for the control group. In all the treated groups, the scar size decreased significantly after 5 weeks of MI (*p < 0.001). Notably, the most remarkable reduction was detected in the combination BMSCs/HGF group compared with the HGF alone or BMSCs alone groups (^△^*p* < 0.001).

**Figure 5:**
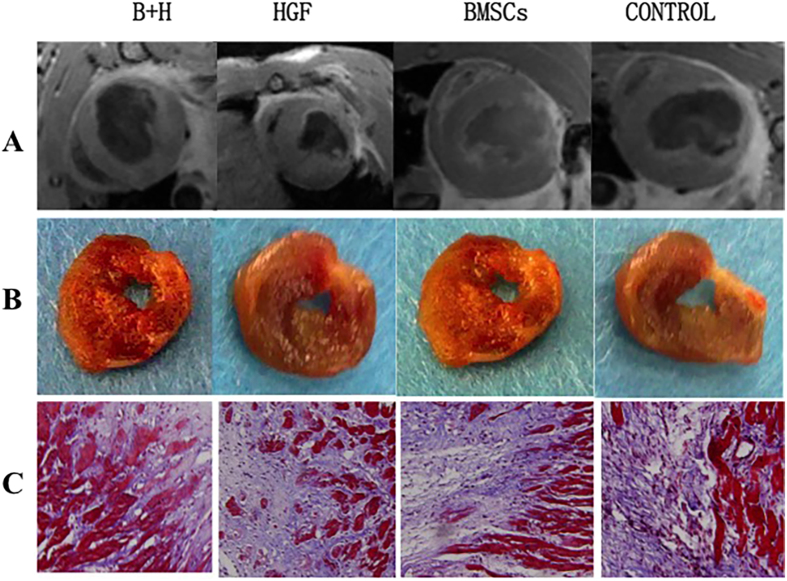
Infarcted areas and levels of myocardial fibrosis were determined by cardiac MR imaging and pathological staining of all the treated and control groups. (**A**) Cardiac MR imaging and (**B**) triphenyltetrazolium chloride (TTC) (x200) detected the LV-infarcted areas, and (**C**) levels of **myocardial fibrosis** were measured using Masson’s trichrome staining (x200).

On delayed contrast-enhanced (DCE) MR imaging, before injection, the areas of acute MI (approximately 20% of the LV) were similar in all groups (*p* = 0.905) one week after MI. Apart from the control group, all treated groups presented significant reductions in infarct size (7.4% to 14.3%) after 3 weeks of infarction. After 5 weeks of MI, in particular, the scar sizes in all treated groups with chronic infarction were significantly reduced compared with acute MI (all *p* < 0.001), whereas the control group had a relatively stable extent of scar from 1 to 5 weeks after MI. Moreover, the scar sizes decreased significantly: 53.0 ± 2.1% in the combination BMSCs/HGF group (*p* < 0.001), 40.4 ± 3.5% in the HGF alone group (*p* < 0.001), 31.7 ± 3.4% in the BMSCs alone group (*p* < 0.001), and 10.4 ± 4.3% in the control group (*p* = 0.036). However, the change in scar size between the groups treated with HGF alone and BMSCs alone was not different (12.4 ± 2.3% *vs.* 13.8 ± 2.9% of LV mass, *p* = 0.982) (Fig. 4). Pathological results indicated that there was no significant difference in the extent of scar tissue measured by TTC staining compared with those detected by MR imaging (*p* = 0.57). To further determine the extent of fibrosis, total myocardial fibrosis within the fibrous infarct region was measured using Masson’s trichrome staining (Fig. 5). The cardiac fibrosis was significantly reduced in all the treated groups compared with the control group (*p* < 0.001), especially in the combination BMSCs/HGF group (*p* < 0.001).

**Figure 6:**
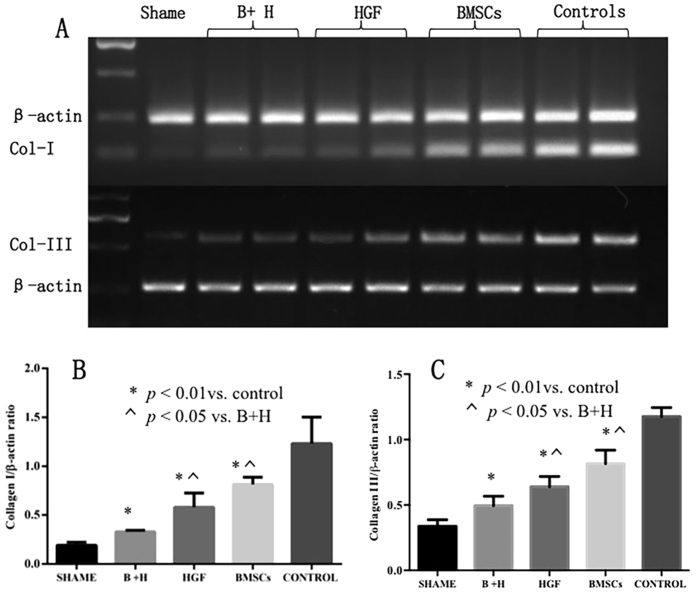
RT-PCR determined type I and type III collagen mRNA expression levels in scars. **(A**) Blots shown are representative of sham and transplanted groups with BMSCs, HGF, BMSCs + HGF (B + H), or phosphate-buffered saline. (**B**,**C**) In all the treated groups, collagen I and III mRNA expression levels were significantly increased compared with the control group (p < 0.001). Importantly, the levels detected in the B + H group were increased compared with those in the BMSCs alone (p < 0.05) and HGF alone groups (p < 0.05). There was no significant difference between the HGF and BMSCs alone groups.

As shown in Fig. 6, RT-PCR was used to determine the expression levels of type I and type III collagen mRNA in the areas of cardiac fibrosis. Compared with the control group, significant reductions were detected in the expression levels of type I (*p* < 0.001) and type III (*p* < 0.001) collagen mRNA in all treated groups. Most importantly, the group treated with BMSCs/HGF showed significantly reduced cardiac fibrosis compared with BMSCs alone or HGF alone both in terms of type I (*p* < 0.001) and type III (*p* < 0.001) collagen expression. Although there was no significant difference, it is notable that the cardiac fibrosis inhabitation in the HGF group is better than that in BMSCs alone, as shown by both Masson’s trichrome staining and RT-PCR analysis.

### Stimulating Angiogenesis and Cardiomyocyte Regeneration

**Figure 7:**
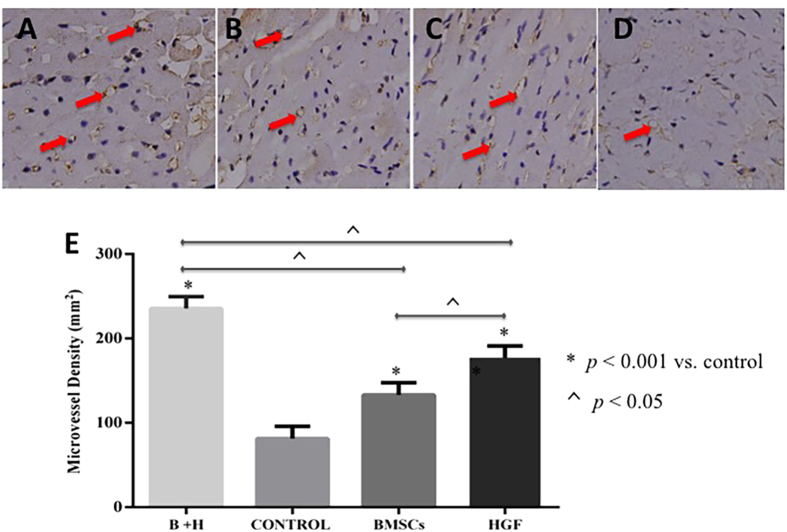
Microvessel densities were demonstrated and compared in all the treated and control groups. Representative micrographs (x400) illustrating peri-infarct sections of hearts transplanted with BMSCs + HGF (**A**), HGF (**B**), BMSCs (**C**), and phosphate-buffered saline (**D**) stained with an antibody against CD34 to visualize and count the microvessel densities (red colour arrows). (**E**) The microvessel densities were significantly increased in the treated groups compared with the saline group (*p* < 0.001). Notably, there were significantly more vessels, representing increased angiogenesis, in the BMSCs + HGF group than in the other treated groups (*p* < 0.05  vs. BMSCs alone and HGF alone). Interestingly, the microvessel density of the HGF group was significantly increased compared with the BMSCs alone group (*p* < 0.05).

The overall difference in microvessel density (MVD) values between the treated groups and the control group confirmed that the implanted BMSCs and/or HGF stimulated angiogenesis after MI (*p* < 0.001 vs. control). In particular, the highest MVD was detected in the combination BMSCs/HGF treated group (all *p* < 0.05), and the MVD of the group treated with HGF alone was significantly greater than that of the group treated with BMSCs alone (*p* < 0.05) (Fig. 7).

**Figure 8:**
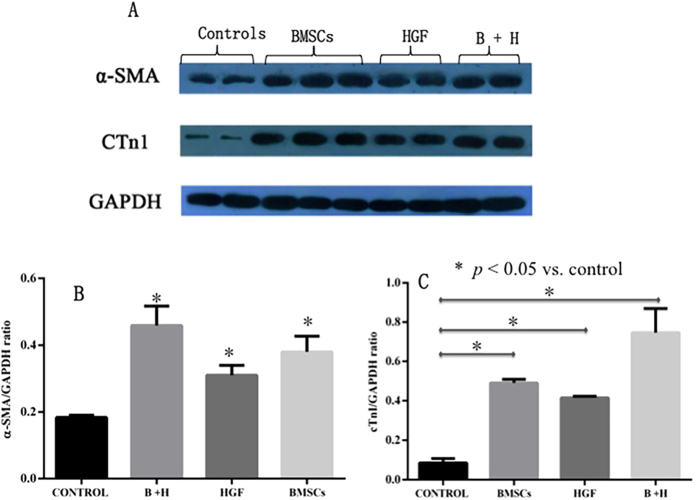
Myocardial a-SMA and cTnI protein levels were evaluated and compared. (**A**) Western blot analysis was used to detect the myocardial a-SMA and cTnI protein levels. After therapy, a-SMA (**B**) and cTnI (**C**) protein expression levels were significantly increased in the treated group compared with the control group (all *p* < 0.05 vs. control).

Myocardial protein levels of a-SMA and cTnI were evaluated via Western blotting. Four weeks after cell transplantation, a-SMA and cTnI expression levels were significantly increased in the treated groups (all *p* < 0.05 vs. control) (Fig. 8).

## Discussion

As a pathological entity of extracellular matrix remodelling after infarction injury, ischaemic myocardial fibrosis often leads to increased myocardial stiffness, which may contribute to LV dysfunction, ventricular tachyarrhythmias, heart failure, and even sudden cardiac death^1^,^2^,^3^,^4^,^5^,^15^,^16^. As a new therapeutic target for heart diseases, myocardial fibrosis has important prognostic implications^2^. To improve patient outcomes, it is clinically critical to attenuate myocardial fibrosis. Therefore, noninvasive detection, assessment, and monitoring of myocardial fibrosis would be valuable in the case of MI. Such information may be helpful for guiding treatment decisions and impacting prognosis^2^,^3^,^11^.

Our data suggested that transfection of HGF into BMSCs for MI treatment could significantly improve LV function, stimulate microvessel angiogenesis, enhance cardiomyocyte regeneration, and inhibit myocardial fibrosis compared with BMSCs or HGF injection alone. With regard to the therapeutic benefits of BMSC implantation, numerous previous studies^8^,^14^,^17^ have demonstrated that cardiomyocyte regeneration cannot be directly attributed to BMSCs. Most importantly, the paracrine therapeutic effects of BMSCs are beneficial for promoting angiogenesis and intervening scar formation at the border of the infarcted myocardium through the secretion of protective factors, such as vascular endothelial growth factor, basic fibroblast growth factor, and hepatocyte growth factor^5^,^8^,^14^,^18^,^19^. Consistent with these above-mentioned studies, our study indicates that BMSC injection alone could promote a-SMA and cTnI expression to some extent. The current study revealed that HGF alone showed a slightly increased promotion of microvessel angiogenesis and reduced myocardial fibrosis compared with BMSCs alone, but these differences did not reach statistical significance. However, we believe that these improvements can play a key role in the treatment of MI. As shown in previous reports^5^,^9^, HGF can promote angiogenesis, which may be beneficial for revascularization of the ischaemic myocardium and could contribute to salvaging hibernating native cardiomyocytes after MI. In addition, HGF exerts anti-apoptotic actions by upregulating *Bcl*-xL and *Bcl*-2 expression^5^. Moreover, HGF can further induce cardiac stem cell activation and migration to activate the endogenous cardiac repair mechanism^10^. Specifically, HGF markedly attenuates myocardial fibrosis by decreasing matrix metalloprotease 2 and 9 expression levels and by inhibiting the formation of collagens I and III^20^. For the ideal cell-based therapeutic strategy, accumulated evidence has demonstrated that BMSCs combined with HGF can improve both structural and functional parameters for ischaemic heart failure^5^,^8^,^9^,^10^,^20^. Our data showed that myocardial fibrosis can be markedly reduced by the combined therapeutic strategies. The therapeutic benefit of combined treatment could be due to the direct inhibitory action of myocardial fibrosis by both HGF and BMSC transplantation. Alternatively, synergistic inhibition was produced by the circle of ventricular remodelling, microvessel angiogenesis, and cardiomyocyte regeneration.

To reduce the injury of chest re-opening for injection of BMSCs in MI animals, we used echocardiography to guide the cell transplantations. As a sensitive and specific imaging modality, bioluminescence imaging was used to dynamically monitor the survival rate of the BMSCs in the myocardium after intramyocardial injection. In contrast to conventional histological staining at different time points, we can noninvasively track BMSCs *in vivo* to decrease the need to sacrifice animals. Similar to previous studies^19^,^20^, in the present study, BLI/D-Luc signals were detectable immediately after injection and began to dissipate approximately 1 week after injection. In practice, MSC longevity is not consistent in different reports. For example, Wang *et al*.^19^ demonstrated the longest survival to date, beyond 50 days. Although the BLI is a sensitive method to track the survival status of BMSCs, the bioluminescence signal intensity is dynamically correlated with the original number of injected cells and not automatically equivalent to cell survival due to its intrinsic limitations. Additionally, BLI cannot be used to accurately locate the transplanted BMSCs due to detection limits.

To target these BMSCs, the distribution of BMSCs labelled with PEI2k-SPIO was traced dynamically using molecular MR imaging due to its high spatial resolution. Apart from dynamic cell tracking, we also performed multiple MR imaging examinations to quantitatively assess LV function and myocardial fibrosis to evaluate the therapeutic impact of cell transplantation. Our study found that LVEF values were increased approximately 2-fold in the BMSCs/HGF group compared with the injection of BMSCs alone for infarcted myocardium. More importantly, myocardial fibrosis has been noninvasively and quantitatively assessed and has been previously confirmed to be a powerful independent predictor of mortality in those patients^2^,^3^. Thus, active intervention, noninvasive monitoring and quantitative assessment of ischaemic myocardial fibrosis are actually necessary. Currently, LGE of MR imaging has emerged as a powerful modality for the quantitative assessment local or diffuse myocardial fibrosis^2^,^15^. In our study, LGE correlated well with the pathological results and demonstrated that the BMSCs/HGF group experienced significantly reduced cardiac fibrosis compared with BMSCs alone or HGF alone in terms of both types I and III collagen. However, LGE can detect fibrosis at only very advanced stages, so early detection of myocardial fibrosis will likely require the development of a molecular targeting contrast agent with increased sensitivity and specificity. For instance, Helm *et al*.^14^ have developed a new collagen-specific MR imaging contrast agent for target imaging type I collagen in myocardial scar, which may be a promising direction for further investigation.

Our study had several limitations. Firstly, a major limitation of current study is that we did not assess the status of new capillaries in the infarcted myocardium with perfusion MR imaging. MR imaging was used in this study to dynamically trace the distribution of BMSCs and to noninvasively monitor the therapeutic effects because of its excellent spatial resolution. During the course of the study, cardiac function, MI extent and fibrosis size can also be assessed quantitatively using a series of MR imaging techniques. Our histopathological findings supported the hypothesis that BMSCs and HGF therapy can stimulate the neovascularization in peri-infarcted and infarcted regions, which can be visualized noninvasively with perfusion MR imaging, as has been previously reported^21^. Secondly, BMSCs have been usually considered safe after transplantation and have been widely tested and proven efficacious in preclinical and clinical studies for cardiovascular diseases, however, the risk of tumor formation still raises caution for human clinical practice^22^. At the present study, the tumor markers express or up-regulated have not been tested and monitored after the treatment. Finally, the expression levels of types I and III collagen have been tested in our study for determining cardiac fibrosis. Besides collagen, other markers or any cytokines of cardiac fibrosis such as matrix metalloproteinases (MMPs) could help to scientifically prove cardiac fibrosis, which will be investigated in our future study.

## Conclusions

Under echocardiography guidance, intramyocardial injection of transfected HGF with BMSCs can enhance cell survival, improve cardiac function, stimulate angiogenesis, and reduce myocardial fibrosis in a post-MI rat model. Moreover, the capacity to rapidly, dynamically monitor treatment processes and simply, accurately quantify the therapeutic effects with multimodal molecular imaging modalities allows a more specific and timely strategy to be established in the regenerative treatment for attenuating myocardial fibrosis.

## Materials and Methods

### Animal Care and Myocardial Infarction Models

All animal protocols and imaging procedures were performed in accordance with protocols approved by the Sichuan University animal care and use committee. For all procedures, rats were anaesthetized by means of an intraperitoneal injection of pentobarbital sodium (80 μg per gram of body weight) and maintained with inhaled isoflurane (1 vol% in oxygen).

To establish the myocardial infarction model, male Sprague-Dawley rats (170–210 g body weight) underwent surgery to permanently occlude the left anterior descending coronary arteries. MI was successfully induced in 28 male Sprague-Dawley rats, which were randomly assigned to 4 groups (7 rats in each group). There were 3 treated groups, those receiving HGF-infected BMSCs (HGF/BMSCs), HGF alone, or BMSCs alone, and one control group receiving placebo. In the HGF/BMSCs group, 2 animals died before the cell injection, so only 5 animals were included in that group. In addition, a sham group consisted of 7 rats that underwent thoracotomy and cardiac exposure but without coronary artery ligation.

### BMSC Culture, Transfection, Labelling and Intramyocardial Cell Injection

BMSCs were obtained, isolated, and expanded as described previously^17^. Lentivector-mediated human HGF transfection and the expression of HGF in BMSCs were performed and verified according to previously reported methods^23^. As described in our previous methods^24^,^25^, the 3^rd^ passage of BMSCs was double-labelled with luciferase and polyethylenimine 2k superparamagnetic iron oxide (PEI2k -SPIO) particles. After labelling, the viability, proliferation, and differentiation capacity of the labelled BMSCs were verified. To reduce immunological rejection, 7 days after MI, echocardiography was used to identify the infarct area and to guide the injection needle. Before injection, the BMSCs were thawed, washed and resuspended in phosphate-buffered saline (PBS). Then, 2 × 10^7^ HGF/BMSCs (10^6^/100 μl), 2 × 10^7^ BMSCs alone, or HGF (2 × 10^6^ TU/100 μl) was suspended in 0.5 ml PBS and was injected into the border zone of infarcted heart tissue. PBS alone (0.5 ml) was used as the placebo injection and was administered to the control group.

### Serial Multimodal Molecular Imaging

#### Echocardiography

Prior to MI procedures, a transthoracic echocardiograph was performed in animals using a Vivid 7 echocardiograph (General Electric-Vingmed, Horten, Norway) equipped with a dedicated small animal probe (i13L, 10 mHZ). Two-dimensional images and M-mode tracings were used to obtain anatomical parameters and global function parameters. Under echocardiograph guidance, the depth puncture and injection point were measured and monitored by a radiologist experienced in ultrasonic intervention during injection. After injection, the distribution of BMSCs and placebo in the myocardial muscle were verified using echocardiography.

#### Bioluminescence imaging

To obtain bioluminescence images, an IVIS spectrum PerkinElmer system (Xenogen, Hopkinton, Mass) with a standard CCD camera and dedicated software (Living ImageTM, Xenogen) was used to collect the serial images. After the substrate D-Luciferin (375 mg/kg) was intraperitoneally injected five minutes before imaging, as described in previous studies^20^,^26^, the BLI/D-Luc signals in animals were imaged after 40 minutes, 3, 5, 7, and 9 days.

#### MR Imaging

A 7.0-T MR scanner (BioSpec *In-vivo* MR Spectroscopy Imaging System, Bruker, Germany) was used to verify and track the labelled BMSCs with PEI2k-SPIO *in vitro* and *in vivo* and to further assess the infarct size, global function and myocardial fibrosis after 1, 3, and 5 weeks of MI. After scout imaging, left ventricular long-axis two- and four-chamber images were acquired using a segmented ECG-gated FLASH black-blood sequence (TR/TE, 210.3/2.1 ms; flip angle, 15°; slice thickness, 1 mm with no gap; field of view, 40 × 40 mm; matrix size 192 × 192). For global function analysis, short-axis images from the base to the apex were acquired using steady-state free-precession cine sequences (TR/TE, 5.2/2.0 ms; flip angle, 10°; slice thickness, 1 mm with no gap). To obtain late gadolinium enhancement (LGE) images, a type of gadolinium chelate contrast agent (gadobenate dimeglumine (MultiHance), 0.5 mmol/ml; Bracco, Milan, Italy) was intravenously injected at a dose of 0.2 ml/kg body weight. After a 20-minute injection, short-axis LGE images were acquired to define infarcted myocardium and myocardial fibrosis using a FLASH sequence (TR/TE, 210.3/2.1 ms; flip angle, 15°; slice thickness, 1 mm with no gap).

### Imaging Analysis

Two experienced radiologists reviewed and analysed the images without any therapeutic information. Any discrepancies in their interpretations were resolved by discussion until a consensus was reached. To determine the effect of combination HGF and BMSC therapy for MI, global function, infarct size and fibrosis were assessed using dedicated software (CMR^42^, Circle Cardiovascular Imaging Inc., Canada). On the LGE images, the scar of ischaemic myocardium was defined as a region with an image intensity greater than 2.0 standard deviations above the mean image intensity in the same image. As previously described^15^,^27^, the extent of scarred myocardium was determined on each of the short-axis images, and scar volume was calculated and expressed as a percentage of LV mass (%LV).

### Postmortem Analysis

At the end of the 5 weeks after MI, animals were humanely killed, and hearts were excised for pathological and immunohistochemical analyses. The representative tissue sections of infarcted myocardium that were comparable to MR imaging transverse sections were visually matched on the basis of anatomical landmarks. Investigators without information of the experimental groups blindly analysed all the pathological and immunohistochemical results.

#### Pathological staining

To measure myocardial infarct size, hearts were sectioned transaxially and incubated in 1% 2,3,5-triphenyltetrazolium chloride (TTC) (Sigma, St Louis, MO, USA). After staining, viable myocardium presented as a deep red area, whereas the infarcted area appeared as a yellow-white area that was unstained by TTC. The true infarct size on the TTC-stained slices was measured directly and was calculated as the ratio (%) of cumulative infarct area to the entire LV area using ImageJ software (Version 1.46, National Institutes of Health, USA). To measure myocardial fibrosis, Masson’s trichrome stain was used for each paraffin-embedded transaxial LV section as previously described^4^. In the Masson trichrome-stained sections, myocardial cells appeared red, whereas fibrillar collagen was identified by its blue-coloured appearance. The total levels of fibrosis in the areas of the fibrous infarct regions were measured with ImageJ software and expressed as percentages of the total LV^28^.

#### Immunohistochemical analysis

To determine the microvessel density (MVD) of the border of the injected sites, myocardial tissue sections were prepared and stained as previously described^29^. Briefly, mouse monoclonal antibodies (Dako, Carpinteria, CA, USA) were used to highlight the endothelial antigen CD34 using routine immunoperoxidase methods. Border zones around the injection site of the infarction were taken to count using an Olympus microscope. When counting, an individual microvessel was defined as any one brown-staining endothelial cell or cell cluster that was obviously different from adjacent microvessels, peripheral tissues and connective tissues. The microvessel density was counted in each of the five random sampling areas at high magnification, as described in a previous report^30^.

#### Real-time PCR

Quantitative Real-time polymerase chain reaction (qRT-PCR) detection was performed to determine the expression levels of fibrillar collagen types I and III (Col-I and Col-III) at the border of the infarct region. Primers were synthesized as follows: forward primer 5′-CCAGTTCGAGTATGGAAGCGA-3′ and reverse primer 5′-AGGTGATGTTCTGGG-3′ for Col-I; and forward primer TTG GAG GTG AAA AGT CTG GCG GCT-3′ and reverse primer TGC AGC CTT GGT TAG GAT CAA CCC-3′ for Col-III. Fluorescent quantitative RT-PCR was performed using an ABI Prism 2720 instrument (Applied Biosystems, USA). RNA was quantified by measuring the absorbance at OD260. The purity and integrity of the RNA were evaluated via gel electrophoresis. For the data analysis, Col-I and Col-III mRNA expression levels were standardized against the internal reference β-actin. Based on the “delta-delta method”, the RNA levels were expressed as ratios to compare the relative expression results among the sham and different treated groups.

#### Western blotting

To assess the effect of the combined cell and gene therapy on the stimulation of angiogenesis and cardiomyocyte regeneration, western blotting for α-smooth muscle actin (a-SMA) and cardiac troponin I (cTnI) was performed 5 weeks after MI. Total protein from myocardial tissues on the border of the infarct region was extracted and separated via sodium dodecyl-sulfate-polyacrylamide gel electrophoresis (SDS-PAGE). The bicinchoninic acid (BCA) assay (Pierce, Rockford, USA) was used to determine the protein concentrations of a-SMA and cTnI. The ratios of the band intensities to that of glyceraldehyde-3-phosphate dehydrogenase (GAPDH) were obtained to quantify the relative protein expression levels and to control for sampling errors.

### Statistical Analysis

All values are expressed as the means ± standard deviations (SD). Statistical analysis was performed using SPSS software (version 16.0; SPSS; Chicago, IL) and GraphPad Prism (version 6.01; GraphPad Software Inc., La Jolla, CA). Differences between groups were determined by analysis of variance (ANOVA), and a Bonferroni correction was used for multiple comparisons between groups. A level of *p* < 0.05 was considered statistically significant.

### Ethical approval

All applicable international, national, and/or institutional guidelines for the care and use of animals were followed. This article does not describe any studies with human participants performed by any of the authors.

## Additional Information

**How to cite this article**: Chen, H. *et al*. Mesenchymal Stem Cells Combined with Hepatocyte Growth Factor Therapy for Attenuating Ischaemic Myocardial Fibrosis: Assessment using Multimodal Molecular Imaging. *Sci. Rep.* **6**, 33700; doi: 10.1038/srep33700 (2016).
